# A Case Report of Refractory Hyperlactatemia Secondary to Thiamine Deficiency

**DOI:** 10.7759/cureus.60760

**Published:** 2024-05-21

**Authors:** Austin Rahman, Nicolette Casarcia, John Frederick

**Affiliations:** 1 Emergency Medicine, Lake Erie College of Osteopathic Medicine, Bradenton, USA; 2 Internal Medicine, Lake Erie College of Osteopathic Medicine, Bradenton, USA; 3 Internal Medicine, AdventHealth Waterman, Tavares, USA

**Keywords:** ldh deficiency, vasculitis, lactate, lactate dehydrogenase (ldh), lupus, sle, thiamine

## Abstract

Thiamine is an essential water-soluble vitamin that must be obtained through diet. This vitamin is crucial for various biochemical reactions and is vital for aerobic metabolism. When individuals are deficient in thiamine, which can be due to hypermetabolism (such as in inflammation, ischemia, or malnutrition, among other reasons), anaerobic metabolism may be utilized to maintain energy needs. Such chemical processes produce lactic acid. Excess lactic acid can cause various clinical signs and symptoms, though lactate dehydrogenase (LDH) can typically break down this compound. The following case presents a very unusual instance where a 51-year-old Caucasian woman presented with the chief complaint of ongoing and severe abdominal pain. After an extensive work-up ruling out numerous diagnoses and an eight-day hospital stay, it was believed that she may be suffering from hyperlactatemia secondary to thiamine deficiency, as she improved significantly after administration of this vitamin. It was thought that this was likely due to her previous systemic lupus erythematosus (SLE) diagnosis, vasculitis, chronic inflammation, and a hypermetabolic state, in addition to concurrent LDH malfunction.

## Introduction

Lactate, or lactic acid, is a naturally occurring molecule in the human body. It is often used as a clinical biomarker for acute inflammation or ischemia, as it is often produced during anaerobic metabolism [1]. Lactate dehydrogenase (LDH) is an enzyme associated with lactate breakdown during anaerobic metabolism. The exact reasoning is due to the biochemical pathways within cells used in response to oxygen deprivation, which becomes prominent when inflammation is evident [1]. Although lactate levels are utilized in the clinical setting and have a high sensitivity to inflammation, it is not specific in determining a specific pathology, which is a significant drawback [2]. The clinical response to therapeutic intervention is frequently measured by lactate levels, and clinicians may use this marker to determine whether a treatment change is necessary [2]. LDH dysfunction, which can lead to elevated lactate or hyperlactatemia, is not commonly seen in documented pathology [3]. Various other ailments can mask underlying hyperlactatemia [3]. A key molecule used by the enzyme LDH is thiamine [3]. This case study highlights a proposed occurrence of thiamine-induced LDH deficiency.

## Case presentation

A 51-year-old Caucasian female with a past medical history of Factor V Leiden, systemic lupus erythematosus (SLE), avascular necrosis of the right hip, deep venous thrombosis, pulmonary emboli, inferior vena cava filter placement, and an embolic cerebral infarction presented to the emergency department with a complaint of severe abdominal pain. Utilizing the systemic inflammatory response syndrome (SIRS) criteria, the patient was admitted to the hospital with a heart rate of 104 beats per minute (bpm) and greater than 22 respirations per minute. A lactate panel was also collected and showed an elevated level of 3.10 mmol/L. The patient also had an elevated body mass index at 44. All other tests ordered, including a complete blood count (CBC) (Table 1) and comprehensive metabolic panel (CMP) (Table 2), were within range or with no significant abnormality.

**Table 1:** Complete Blood Count

Result	Value	Reference Range
White Blood Cells	6.97	3.30-10.60 10^3/uL
Red Blood Cells	4.61	3.42-5.40 10^3/uL
Hemoglobin	12.1	11.8-16.0 g/dL
Hematocrit	38.2	35-47%
Mean Corpuscular Volume	82.9	80-99 fL
Mean Corpuscular Hemoglobin	26.2	27-33 pg
Mean Corpuscular Hemoglobin Concentration	31.7	33-36 g/dL
Red Cell Distribution Width	16.4	10.3-13.8%
Platelet Count	156	148-426 10^3/uL
Mean Platelet Volume	10.5	7.0-10.5 fL
Neutrophils %	75.2	50.0-70.0%
Lymphocyte %	17.4	21.0-45.0%
Monocytes %	6.9	1.0-15.0%
Eosinophils %	0.1	0.0-5.0%
Basophils %	0.0	0.0-3.0%
Neutrophils Absolute	5.24	1.3-7.2 10^3/uL
Lymphocytes Absolute	1.21	1.0-4.9 10^3/uL
Monocytes Absolute	0.48	0.3-1.0 10^3/uL
Eosinophils Absolute	0.01	0.0-4.0 10^3/uL
Basophils Absolute	0.00	0.0-0.3 10^3/uL
Nucleated Red Blood Cell Concentration %	0.0	0%
Nucleated Red Blood Cell Concentration Absolute	0.0	0.00-0.30 10^3/uL
Immature Granulocytes %	0.4	0-0.6%
Immature Granulocytes Absolute	0.03	10^3/uL

**Table 2:** Comprehensive Metabolic Panel

Result	Value	Reference Range
Sodium	139	136-145 mmol/L
Potassium	3.8	3.2-5.1 mmol/L
Chloride	103	101-111 mmol/L
Carbon Dioxide	24.0	22.0-30.0 mmol/L
Anion Gap	12	3-11 mmol/L
Blood Urea Nitrogen (BUN)	12.0	6.0-20.0 mg/dL
Creatinine	0.70	0.40-1.40 mg/dL
BUN/Creatinine Ratio	17.1	10:1-20:1
Glucose	135	74-100 mg/dL
Calcium	8.6	8.0-10.2 mg/dL
Aspartate Aminotransferase* (*AST)	12	15-41 U/L
Alanine Transaminase (ALT)	24	6-48 U/L
Alkaline Phosphatase	107	38-126 U/L
Protein, Total	6.3	6.6-8.7 g/dL
Albumin	3.60	3.50-5.20 g/dL
Globulin	2.7	2.0-3.5 g/dL
Albumin/Globulin Ratio	1.3	1.0-2.5
Bilirubin, Total	0.40	0.20-1.20 mg/dL
Osmolality Calculation	270	mosm/kg
Estimated Glomerular Filtration Rate (eGFR)	104.9	mL/min

After admission to the hospital, the patient continued to complain of post-prandial abdominal pain, nausea, and vomiting. Soon after admission, the patient had a blood pressure of 126/65 mmHg, pulse of 70 bpm, respiratory rate of 19, and temperature of 98.4°F, with an oxygen saturation of 93% on room air. The patient was given 4 mg of ondansetron, 4 mg of morphine, and 15 mg of ketorolac for symptom management. Upon admission to the hospital, the patient’s uptrending lactate levels were of primary concern (Table 3). The patient had a prior admission to the hospital for a different chief complaint, in which baseline lactate levels were established and used as a comparison (Table 3). Vasculitis was determined to be the suspected diagnosis, and intravenous (IV) fluids of 0.9% saline, imaging, and stool studies were ordered. Various imaging studies, which were confirmed by the radiologist, determined the patient to have benign findings with residual attenuation due to recovering from a previous coronavirus (COVID-19) infection. 

**Table 3:** Lactate Levels + (plus symbol): Prior lactate levels that were drawn from past hospital admissions unrelated to current hospital admission * (asterisk): Decreased lactate levels were noted, but significant abdominal and clinical symptoms were still present, and lab error was also suspected on this day Lactate blood levels were drawn every six hours

Reference Range	15 Days Prior^+^	16 Days Prior^+^	Day 1	Day 2	Day 3	Day 4	Day 5	Day 6*	Day 7	Day 8
0.50-2.20 mmol/L	1.10	1.20	2.40, 2.20, 3.10	2.90, 3.30, 3.40	2.30, 2.60, 4.60	2.70, 2.50, 4.20	3.10, 3.30, 2.60	2.00	5.70, 2.70, 2.60	1.80

On the third day of hospitalization, her hyperlactatemia persisted, with the patient complaining of continued severe abdominal pain. A repeat CT of the abdomen and pelvis with IV contrast was ordered, with a consultation made with gastroenterology and general surgery for possible mesenteric ischemia. Imaging the abdomen exhibited benign findings, including no segmental bowel dilatation, air-fluid levels, or lumen narrowing. Due to these findings, an MRI angiogram of the abdomen was utilized to rule out possible arterial insufficiency of the bowel. Gastroenterology and general surgery consult agreed that the hyperlactatemia was likely to be present secondary to vasculitis. Radiology consultation was also appreciated, and the risk of continued imaging was believed to outweigh the benefits associated with the problem at hand. It was thought to be more likely that the patient had an underlying issue that could not be assessed by direct imaging. 

On the fifth day of admission, a rheumatology consult was made, and a suspected diagnosis of an SLE flare-up that may be causing bowel vasculitis was considered. The rheumatologist recommended high systemic steroids (Solu-Medrol, or methylprednisolone, one gram for three days with continued extensive IV fluids, followed by a steroid taper) for treating this individual. Cyclophosphamide was discussed as a potential treatment option, if the steroid trial fails. Methylprednisolone was given the next day (day 6) with a complete diet. 

Interestingly, the patient’s lactate levels initially decreased slightly on the sixth day of admission. However, the patient complained of persistent severe abdominal pain that continued with food intake. 

On admission day 7, a significant increase was noted regarding the patient’s lactate levels, and her symptoms persisted. With mainstay clinical avenues exhausted, the attending hospitalist made a clinical decision to attempt to treat the patient with 100 mg of IV thiamine. Upon administration of this vitamin, on day 8, the patient's lactate levels stabilized (Table 1), her abdominal pain ceased, and she could tolerate a regular diet. At that time, she was determined to be clinically stable. The patient was successfully discharged with a prescription of 100 mg of oral thiamine daily. A consultation was made to the state academic center for referral and follow-up, to which the patient agreed.

## Discussion

Clinical symptoms and patient history are the cornerstones of how physicians tackle the manifestations of diseases. However, less common pathologies may mask such presentations. This patient's medical history was a significant factor in suspecting vasculitis as the prime culprit. As such, proper consultations and testing were done to rule out this disease process. Thiamine deficiency is often not associated with elevated lactic acid [3]. It should also be noted that a possible lab error could have been a culprit in the decrease in lactate on admission day 6. Clinical symptoms were still significant, in addition to a high rise in lactate the day after. Medical literature often describes thiamine deficiency in the context of patients with alcohol use disorder who are suffering from Wernicke's encephalopathy or a manifestation of “beri-beri” [3]. Thiamine is a water-soluble vitamin B complex; thus, diuretics or dehydration may lead to deficiencies [4]. Our patient was not on any diuretics and was on continuous IV fluids. Additionally, thiamine deficiency may present in those who are malnourished or have malabsorptive diseases. Regardless of these commonplace manifestations, medical literature does show that thiamine deficiency has a role in causing elevated lactic acid levels. After looking at prior case reports, thiamine deficiency is noted in the medical literature as a secondary cause of elevated lactate [5]. After a thorough investigation, a last attempt was made to try and alleviate the patient’s symptoms before transferring her to an equipped academic hospital. Thus, thiamine was given. A great fear was that the patient would deteriorate based on the prolonged stay in addition to her elevated lactate levels. The prolonged patient stay complicated the situation, as it is known that increased hospital stays are associated with more hospital-acquired co-morbidities [6].

An interesting question is why the patient had thiamine deficiency, as the patient denied any change in diet, medication, or alcohol use. Interestingly, a case report demonstrates Wernicke's encephalopathy, caused by thiamine deficiency, in another SLE patient [7,8]. Therefore, there may be a correlation between thiamine deficiency and SLE. However, more primary research and confounding variables must be excluded before significant clinical assumptions or correlations can be made. 

Thiamine is an essential vitamin in metabolizing carbohydrates, proteins, and fats. Those deficient may have serious side effects, such as in the case presented. Due to the inability of humans to synthesize thiamine, we depend on obtaining this water-soluble vitamin through food. It is recommended that men consume 1.2 mg of thiamine daily, women consume 1.1 mg/day, pregnant women consume 1.4 mg/day, and lactating women consume 1.5 mg/day. There are no known effects of high thiamine supplementation due to water solubility, and therefore, there is no upper limit of thiamine intake [9]. When deficient, patients may experience weight loss, muscle weakness, peripheral neuropathy, decreased immunity, confusion, or even memory loss [9,10]. Thiamine can be converted to thiamine diphosphate, in addition to many other products, which are utilized in the citric acid cycle, the pentose phosphate pathway, and aerobic glycolysis. These biochemical pathways are crucial for carbohydrate, fat, and protein metabolism, ultimately providing energy for cell and organ function. Currently, there is no universally accepted biomarker of thiamine levels in humans. Thiamine diphosphate may be obtained in whole blood, though this is often difficult to do, as the specimen must be appropriately handled [9]. When phosphorylated, thiamine can be stored in the heart, liver, kidneys, and brain. However, stores can be depleted in about two to three weeks. Hyper-metabolic states, such as infections, inflammation, diabetes, alcohol withdrawal, and seizures, may decrease these stores even more rapidly [10]. In this case, it is believed that this patient's SLE diagnosis and possible vasculitis may have induced a hypermetabolic state, potentially utilizing all of her thiamine stores. As stated, thiamine derivatives, such as thiamine diphosphate, play an important role in glycolysis, which aids in breaking down carbohydrates, in particular. Thiamine is a cofactor for pyruvate dehydrogenase, which allows pyruvate to relocate into the mitochondria. Downstream pathways allow acetyl-coenzyme A (acetyl-CoA) to enter the citric acid cycle. When pyruvate cannot enter the mitochondria, which happens in thiamine-deficient states, excess pyruvate may be converted into lactate for anaerobic energy usage [9,10]. When resources are unavailable for aerobic metabolism to continue, such as chronic or acute inflammatory reactions, excessive muscle usage, ischemia, or infection, lactate builds up. Unfortunately, elevated lactate levels can result in pain, which may be located in various regions, nausea, cramping, weakness, or tachypnea [11,12]. LDH is then able to break down excess lactate. But to worsen matters, the patient’s LDH was suspected not to be working correctly due to her vitamin deficiency, again increasing lactic acid levels. Through extensive clinical research, the patient's past medical history, and the use of multiple sources, including numerous physician consults, as well as the spontaneous resolution of the clinical symptoms once thiamine was administered, we believe this to be the most likely answer to our clinical questions. In addition, it was discovered that a very similar case was presented in a 31-year-old male who complained of severe epigastric pain, nausea, and vomiting of a one-week duration. He too had elevated lactate levels, and his symptoms, as well as his hyperlactatemia, ceased after thiamine infusion [13]. 

Furthermore, it should be remarked that measuring thiamine is not routinely done due to the difficulty in the measurement of the water-soluble vitamin [3]. As such, many thiamine deficiency disorders are diagnosed based on patient history and clinical symptoms. Due to the vagueness of an undetermined upper limit and non-standardized testing protocols [3,9], our patient was not directly tested for thiamine deficiency prior to or after administration of thiamine. 

## Conclusions

Although it is essential to consider common pathologies when treating patients, especially those with multiple co-morbidities, keeping rare disorders in the mind’s periphery is crucial. In clinical settings, physicians should be conscious and alert to common pathological manifestations and should be able to use their knowledge and skill base to obtain a lead diagnosis quickly. However, if this approach fails and consultations cannot make a clear clinical decision, it is essential to return to primary literature and explore other avenues. The attending hospitalist's decision to make an effort toward thiamine deficiency was a positive and critical outcome that utilized the principle of diagnosis of exclusion. As stated earlier in this report, thiamine deficiency does not classically cause elevated lactate levels. However, the biochemistry associated with affected pathways makes it an evident cause of the elevation of such markers. We urge physicians to continue to be lifelong learners and to understand, at a deep fundamental level, why specific markers are elevated, correlating patients’ symptomatic complaints to such levels. The importance of diagnosis by exclusion, continuously adapting to new information, and making a clinical diagnosis when common pathologies are ruled out, is an essential asset to a clinician's toolkit. We further urge researchers to conduct studies to obtain an accurate and statistical correlation between SLE and thiamine deficiency. Such clinical pearls can be monumental in disease progression and treatment, bettering the lives of affected patients.
